# Comparing methods for handling missing cost and quality of life data in the Early Endovenous Ablation in Venous Ulceration trial

**DOI:** 10.1186/s12962-022-00351-6

**Published:** 2022-04-07

**Authors:** Modou Diop, David Epstein

**Affiliations:** Department of Applied Economics, University of Granada, Campus de Cartuja, 18071 Granada, Spain

**Keywords:** Longitudinal missing outcome, Repeated measure, Mixed model, Fixed effect, Multiple imputation, Complete-case-analysis, Bayesian parametric approach, Cost-effectiveness analysis

## Abstract

**Objectives:**

This study compares methods for handling missing data to conduct cost-effectiveness analysis in the context of a clinical study.

**Methods:**

Patients in the Early Endovenous Ablation in Venous Ulceration (EVRA) trial had between 1 year and 5.5 years (median 3 years) of follow-up under early or deferred endovenous ablation. This study compares complete-case-analysis (CCA), multiple imputation using linear regression (MILR) and using predictive mean matching (MIPMM), Bayesian parametric approach using the R package missingHE (BPA), repeated measures fixed effect (RMFE) and repeated measures mixed model (RMM). The outcomes were total mean costs and total mean quality-adjusted life years (QALYs) at different time horizons (1 year, 3 years and 5 years).

**Results:**

All methods found no statistically significant difference in cost at the 5% level in all time horizons, and all methods found statistically significantly greater mean QALY at year 1. By year 3, only BPA showed a statistically significant difference in QALY between treatments. Standard errors differed substantially between the methods employed.

**Conclusion:**

CCA can be biased if data are MAR and is wasteful of the data. Hence the results for CCA are likely to be inaccurate. Other methods coincide in suggesting that early intervention is cost-effective at a threshold of £30,000 per QALY 1, 3 and 5 years. However, the variation in the results across the methods does generate some additional methodological uncertainty, underlining the importance of conducting sensitivity analyses using alternative approaches.

**Supplementary Information:**

The online version contains supplementary material available at 10.1186/s12962-022-00351-6.

## Introduction

Missing data occurs when one or all variables are missing for a given subject. This often occurs in longitudinal studies and can particularly be a problem in within-study cost-effectiveness analysis (CEA) because accurate estimates of total mean cost and quality-adjusted life years require full data to be collected on each subject at each follow-up time point [1–3].

This study compares six different methods for handling missing data in a cost-effectiveness analysis comparing early endovenous ablation versus delayed ablation for venous leg ulcer treatment [4]. The original cost-effectiveness analysis employed a repeated measure mixed model (RMM), and reported mean total cost of − £155 (95% CI − £1262 to £953) and mean total QALY of 0.073 (95% CI − 0.06 to 0.20) at 3 years [4]. RMM has been shown to have acceptable properties in simulation studies [5]. However, as missing data are always unknown, it is recommended to conduct sensitivity analyses to see how robust the results are to alternative methods, and this is the primary aim of this paper [1]. This work is unable to demonstrate which approach is “correct” because we do not know the values of the missing data. Nevertheless, this paper provides an interesting case study of “revisional research” in health economics [6], in which the original findings are challenged by employing more extensive methods to assess modelling uncertainty. The methods outlined in this paper may also be useful more generally to investigators wishing to explore the different ways that missing data approaches can be implemented with standard statistical software (STATA or R).

Due to the design of the trial, there was very low loss to follow-up, but considerable item missingness (see “Methods”: “Data”). There are several ways in which the chosen missing data approach might influence the results: different subjects used in the analysis, different number of observations used per subject, different statistical models of the missing data mechanism and the latent correlation between observed and missing observations, or different estimation model to estimate total mean costs and QALYs and the correlation between them [7, 8]. This paper addresses this challenge using six alternative methods: complete case-analysis (CCA) [9–11], multiple imputation by linear regression (MILR), multiple imputation by predictive mean matching (MIPMM) [9, 11–13], repeated measure mixed model (RMM) also known as random effect, repeated measure fixed effect (RMFE) [14], and a Bayesian parametric approach (BPA) using the **selection** model in the R package **missingHE** [15]. All methods assume data are Missing Completely at Random, given covariates (CD-MCAR) or Missing at Random (MAR). Under CD-MCAR, the probability that data are missing only depends on observed baseline covariates, and under MAR, the probability depends only on values of observed outcome data and baseline covariates [1]. The package **missingHE** also provides models to explore missing not at random (MNAR) situations but this is not considered here [5]. Results are estimated over different time horizons (and hence with different quantities of missing data) of 1, 3 and 5 years. In each case we calculate the mean incremental total cost and QALY, standard errors, the incremental cost-effectiveness ratio (ICER) and the cost-effectiveness acceptability curve (CEAC). The focus in this paper is on alternative statistical methods for handling missing data. We do not explore other sources of modelling uncertainty, such as use of different sets of covariates to make predictions or alternative statistical distributions of dependent variables [16, 17].

## Methods

### Data

The Early Endovenous Ablation in Venous Ulceration (EVRA) randomised clinical trial evaluated the cost-effectiveness of early versus deferred endovenous ablation to treat venous leg ulcers. The trial methods and patients are described elsewhere [4]. Briefly, resource use items in hospital, primary and community care and medications related to the treatment of venous ulceration, adverse events or complications were collected by case note review and questionnaires completed at baseline and monthly thereafter up to 1 year, plus one further telephone follow up between October 2018 and March 2019.

The baseline covariates included in all the estimation models were: *TREAT* is treatment randomised (“early” coded as 1 or “delayed” coded as zero). The variable ${WEEK}_{t}$ is the time variable (coded as a set of categorical (dummy or factor) variables) representing the week after randomisation at which data are observed, from t = 0 (baseline) to t = 16 (week 260). *SIZE, AGE* and *DURATION* are the ulcer size (cm^2^), subject’s age (years) and length of time with ulcer (years), respectively, measured at baseline and centred at the means. SITE was coded as a factor variable.

Each item of resource use was multiplied by UK unit costs obtained from published literature, NHS reference costs, and manufacturers’ list prices to calculate overall costs within each of these categories for each patient [4]. The costs for each individual over their follow-up (from randomization to date of censoring for that individual) were assigned or apportioned into discrete time periods, that corresponded to 12 monthly periods during the first year (as follow-ups were monthly) and then yearly periods thereafter. This allowed discounting to be applied (3.5% per year), and facilitated analysis using the MI and mixed model in long format (see below).

EQ-5D-5L was collected at baseline, 6 weeks, 6 months, 12 months, plus one further telephone follow up between October 2018 and March 2019, and a utility index was calculated at each time point using a published tariff [18]. SF-36 was also administered but only up to 1 year, so was not used in this paper.

Patients who died during the study were assigned zero costs and HRQOL thereafter. Code and example data are available in Additional file 1, http://dx.doi.org/10.17632/j8fmdwd4jp.6.

### Missing data

Due to rigorous trial design and conduct procedures [19], there were very few withdrawals or failures to complete questionnaires as planned in the study (see Additional file 1: Table S5). Nevertheless, data are incomplete in this study for two reasons. First, recruitment of the 450 patients into the clinical study across the 20 vascular centres took place between October 2013 and September 2016. The study finalised on March 2019. This “staggered” recruitment into the trial meant that patients had a minimum of 1 years of follow-up and a maximum of 5.5 years (median 3 years).

**Fig. 1:**
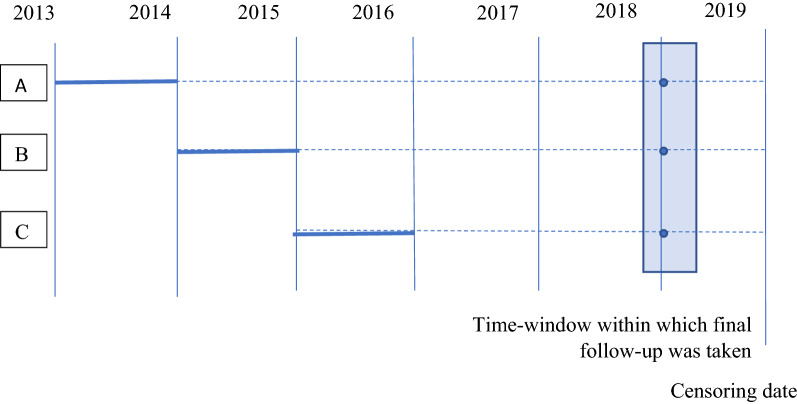
Schematic relation between recruitment date and missing data pattern for 3 hypothetical patients

Second, all patients had regular and periodically scheduled follow-up during the first year after recruitment, but to keep the cost of the research study low, only one further telephone follow-up per patient was conducted. This took place between October 2018 and March 2019. Figure 1 shows how this study design influences the missing data pattern. A patient recruited in 2014 will have complete follow-up during the first year, missing data at years 2, 3 and 4, and one follow-up at 5 years (patient A). A patient recruited in 2015 will have complete follow-up during the first year, missing data at years 2 and 3, one follow-up at year 4, and missing data for year 5. A patient recruited in 2016 (patient C) will have complete follow-up during the first year, missing data at year 2, one follow-up at year 3, and missing data for years 4 and 5. This mainly affected collection of EQ-5D, because in the absence of telephone questionnaire data, most types of resource use and clinical outcomes could be obtained from case-notes.

The pattern of missingness was examined using descriptive statistics and via the linear logistic model of indicators of missing cost and EQ-5D data on treatment allocation and a selection of baseline variables (Eq. 1) [1].

1 $$logit\left({\pi }_{it}\right)= {\gamma }_{1}{TREAT}_{i}+{\gamma }_{2}{DURATION}_{i}+{\gamma }_{3}{AGE}_{i}+{\gamma }_{4}{SIZE}_{i}+{\gamma }_{5}{Site}_{i}+{\gamma }_{6}{WEEK}_{t}$$

where $\pi$ denotes the probability that an observation is missing in individual *i* at time *t.*

Cost-effectiveness analysis was conducted using aggregated data—CCA and BPA—and disaggregated (longitudinal) data—MI, RMM and RMFE. Table 1 summarises the approaches. Further details are also given in Additional file 1

**Table 1:** Overview of approaches employed to handle missing data

	RMM and RMFE	CCA	MILR and MIPMM	BPA
Number of patients included at 3 years	450	44	450	450
Total number of non-missing observations included at 3 years^a^	1929 EQ-5D, 6861 period costs	44 total costs, 44 QALY	450 EQ-5D, 450 period costs	377 total costs, 44 QALY
Format of data as input	Longitudinal	Aggregate	Longitudinal	Aggregate
Statistical model of the missing data	Implicit imputation of missing EQ-5D and period costs	None	Explicit imputation of missing EQ-5D and period costs	Logit model of probability of missingness
How are total costs and QALY over the desired time horizon predicted at individual level?	Not necessary	Not done	Passively in each imputed dataset	Missing total cost and QALY are parameters to estimate
How are mean total incremental costs and QALY over the desired time horizon estimated	Weighted sum of EQ5D and period cost coefficients estimated in the statistical model	Bivariate normal regression	Bivariate normal regression for each imputed dataset, synthesised using Rubin’s rules	Bivariate normal regression
Estimation of standard errors and CEAC	Bootstrap	Parametrically	Parametrically	Parametrically

^a^If aggregate data are used, there will be one observation per patient. If longitudinal data are used, the inputs to the model may consist of several observations per patient

*RMM* repeated measure mixed model, *RMFE* repeated measure fixed effect, *CCA* complete-case-analysis, *MIPMM* multiple imputation using predictive mean matching, *MILR* multiple imputation using linear regression, *BPA* Bayesian parametric approach

### Repeated measure: mixed model and fixed effect

The effects of the events on the HRQOL and costs were computed using repeated measures regression model with the differences between subjects (${\varsigma}_{i}$) modelled as a random effect (RMM) or fixed effects (RMFE) (Eq. 2). The RMFE method eliminates unobserved time-invariant confounders without imposing any additional assumptions on ${\varsigma}_{i}$. The RMM method assumes that unobserved heterogeneity ${\varsigma}_{i}$ is not correlated with other controls [20].

2 $${Y}_{it}={\beta }_{0}+{\beta }_{1}{TREAT}_{i}+{\beta }_{2}{DURATION}_{i}+{\beta }_{3}{AGE}_{i}+{\beta }_{4}{SIZE}_{i}+{\beta }_{5}{Site}_{i}{+ \beta }_{6}{WEEK}_{t}+\delta {TREAT}_{i}*{WEEK}_{t}+{\varsigma}_{i}+{\epsilon }_{it}$$

${Y}_{it}$ is the outcome variable (one model for costs during each period *t* and another for EQ-5D tariff at the end of each period *t*) for each subject *i* at time point *t.* Hence for the model where the dependent variable is cost, ${Y}_{i0}$ is set to be zero for all subjects, ${Y}_{i1}$ is the cost for patient *i* during the first 4 weeks ${Y}_{i2}$ is the cost between the 4th and the 8th week, and so on up to ${Y}_{i12}$ (week 52). After that, the periods are set to be yearly, so that ${Y}_{i13}$ is the cost between week 52 and week 104 (year 2), and so on up to ${Y}_{i16}$ (year 5 or week 260). ${\varsigma}_{i}$ is the random deviation of subject *i*’s mean costs or EQ-5D tariff from the overall mean ${\beta }_{0}$ and ${\epsilon }_{it}$, often called within-subject residual across time, is the random deviation of ${Y}_{it}$ from subject *i*’s mean costs or EQ-5D tariff [21, 22]. ${Y}_{i}$ is the outcome variable for each subject *i.*

In RMM and RMFE estimates of the $\widehat{\delta }$ are a (vector of) coefficients for the interactions between treatment assignment and period number and hence represents the mean incremental cost of early treatment (versus delayed) during period t (in the cost model) or the mean incremental EQ-5D tariff at follow-up time point t (in the EQ-5D model). These analyses were implemented using the **mixed** and **xtreg** command in STATA 15. To estimate total mean incremental cost per patient over a desired time horizon (e.g., 3 years), the relevant period coefficients are simply added up (**lincom**). Thus, for example, where the dependent variable is cost accrued during the preceding period, and ${\widehat{\delta }}_{1}$ is the time-treatment interaction coefficient at 4 weeks (~ month 1), ${\widehat{\delta }}_{2}$ at 8 weeks (~ month 2), and ${\widehat{\delta }}_{3}$ at 13 weeks (month 3), then the difference in total mean incremental cost over the first 3 months is ${\widehat{\delta }}_{1}$ + ${\widehat{\delta }}_{2}$ + ${\widehat{\delta }}_{3}$. To estimate mean total incremental QALY over a given time horizon, the “area under the curve” applying the trapezium rule is calculated. Hence, using the coefficients from the EQ-5D model over the first 3 months (where ${\widehat{\beta }}_{1}$ is the difference in EQ-5D at baseline, ${\widehat{\delta }}_{1}$ at 4 weeks and ${\widehat{\delta }}_{2}$ at 3 months), the estimated mean total incremental QALY over the first 3 months would be $0.5*\left(({\beta }_{1}+{\widehat{\delta }}_{1})*\frac{4}{52}+{(\widehat{\delta }}_{1}+{\widehat{\delta }}_{2})* \frac{9}{52}\right)$).

Uncertainty was estimated by bootstrapping incremental mean costs and QALYs [23] and shown by the cost-effectiveness acceptability curve (CEAC). The bootstrap is used here because in the RMFE and RMM approaches, we run separate regressions for period costs and EQ-5D. In the MI, BPA and CCA approaches, we are able to analytically calculate the variance–covariance matrix using a joint regression of total costs and total QALY (assuming a bivariate normal distribution of the dependent variables) and so could estimate the CEAC parametrically. In the case of the RMM and RMFE models, this option is not available and so the bootstrap presents a pragmatic, numerical solution to this problem.

### Multiple imputation

We implemented MI using three steps. Firstly [24], M imputations (completed datasets) were generated under an imputation model replacing missing values with “plausible” substitutes, based on distribution of the observed data using linear regression (MILR) and predictive mean matching (PMM). The variables included in the imputation models for costs and EQ-5D were treatment, age, duration, site, ulcer size, ethnicity, diabetes, history of deep vein thrombosis, trial leg and Eq. 5d at baseline [25].

This step was performed by multivariate imputation by chained equation (MICE) (also known as fully conditional specification (FCS) [26] or sequential regression multivariate imputation [27]) which is a practical approach to generating imputations based on a set of inter-linked imputation models. The process using MILR begins by choosing the first variable to impute, say costs in the first period ($${Y}_{1}$$). Values for all other variables (both EQ5D at each follow up and period costs) to be imputed were then filled in using a simple rule (simple random sampling with replacement from the observed values). Then, $${Y}_{1}$$ was regressed on all other variables and baseline covariates, and then missing values for $${Y}_{1}$$ were replaced by simulated draws from the corresponding posterior predictive distribution of $${Y}_{1}$$. Then, the process was repeated for the next variable (e.g., $${Y}_{2}),$$ which was regressed on all other variables and using the newly imputed values in $${Y}_{1}$$. Again, missing values in $${Y}_{2}$$ were replaced by draws from the posterior predictive distribution of $${Y}_{2}$$. The process was repeated for all other variables with missing values in turn: this is called a cycle. In order to stabilize the results, the procedure was repeated for 20 cycles to produce a single imputed data set, and the whole procedure was repeated M times to give M imputed data sets [28–31].

A second method for MI, predictive mean matching (PMM) was also used. PMM is an ad hoc method of imputing missing values which combines the standard linear regression and the closest-neighbour imputation approaches. For each missing value ${Y}_{i}$ with covariates ${X}_{i}$, PMM identify k individual with the nearest value of observed ${Y}_{i}$—It uses the linear predictions as a distance measure to form the set of the nearest neighbours (suitable “donor”) consisting of the complete value—, it then randomly draws an imputed value from this set. By drawing from the observed data, PMM preserves the distribution of the observed values in the missing part of the data which makes it more robust than the fully parametric linear approach [32]. Possible donors were set with 10 closest neighbours as suggested in Morris et al. [33].

Step 2 was to perform M = 40 imputations [34], and finally, step 3, the results obtained from the 40 completed-data analyses were combined into a single multiple-imputation result using Rubin’s rules [35]. Analyses were implemented using the **mi** suite of commands in STATA 15.

Monte Carlo Errors (MCE) and the fraction of missing information (FMI) were calculated to indicate the stability of the model. FMI and MCE reflect the variability of MI results across repeated uses of the same imputation procedure and are useful for determining an adequate number of imputations to obtain stable MI results [13].

For each of the m complete datasets, total cost and total QALY over 1 year, 3 years and 5 years for each subject were imputed passively using the same formulas given in the section for repeated measures. The difference between repeated measures and MI being that in the RMM and RMFE approaches, estimates of total mean cost and QALY for the group as a whole were made by linear combination (**lincom**) of the coefficients, while MI imputes a total cost and QALY for each subject, and then proceeds to estimate mean incremental cost and QALYs for the group as a whole using bivariate normal regression (**sureg** in STATA 15). Coefficients from this regression were then combined across the multiple imputed datasets using Rubin’s rules (**mi estimate**) [34]. The bootstrap was not used with MI as this can be complex and time-consuming [36]. Instead, the CEAC was calculated parametrically from the coefficients and covariance matrix of the bivariate normal regression.

### Complete case analysis

Total cost and total QALY were calculated for each individual *i* over the relevant time horizon T (1, 3 or 5 years) (Eq. 3). Any subject with a missing period cost or EQ-5D in one the relevant time horizon was dropped (as total cost and total QALY for individual i at time T cannot be calculated if any period costs or EQ-5D values up to T are missing). A bivariate normal regression was performed at each time horizon for total costs and total QALY (Eq. 3), where ${Y}_{i}$ is a (cost, QALY) pair for individual i. The CEAC was calculated using the bootstrap (parametric estimates were also tried and made no noticeable difference to the results so are not reported).

3 $${Y}_{i}={\beta }_{0}+{{\beta }_{1}{eq5d0}_{i}+ \beta }_{2}{TREAT}_{i}+{\beta }_{3}{DURATION}_{i}+{\beta }_{4}{AGE}_{i}+{\beta }_{5}{SIZE}_{i}+ {\beta }_{6}{SITE}_{i}+{\varepsilon }_{i}$$

### Bayesian parametric approach (BPA)

The dataset for BPA consists of total observed cost and total observed QALY for each individual over the time period of interest (1, 3 or 5 years), along with baseline control variables. Hence one total cost and one total QALY observation per subject are used as dependent variables in the analyses, in the same way as the CCA approach. However, unlike CCA, all individuals are included in the analysis dataset. In BPA each unobserved quantity (total cost or total QALY) in the model is handled as if it were a parameter [37–40].

The BPA was implemented based on Markov Chain Monte Carlo (MCMC) using the R function **selection,** within the **missingHE** package [37]. BPA requires the specification of four models: the first two are the estimation models for the total QALY and total cost variables (*Y*) assuming these data are bivariate normally distributed (as Eq. 3) and the last two are the auxiliary models which are fitted (similarly to Eq. 1) to estimate the probability *Y* is missing using logistic regressions.

The four models include baseline covariates of treatment allocation, ulcer duration, ulcer size, age, and site. And the auxiliary models also include the length of follow-up in the study, as the probability of missingness increases with time since baseline. Non-informative priors were used for the precision of the dependent variables, which were varied from 0.001 to 0.01 in sensitivity analyses. Incremental mean costs and QALY were computed from the estimation models and the CEAC was calculated parametrically from the variance–covariance matrix.

The original cost-effectiveness analysis for the EVRA trial coded SITE as a random effect. The documentation for BPA states that covariates can be included either as fixed or random effects [41], but despite our best efforts and attempting to contact the software authors for advice without reply, we were unable to implement this feature. Hence in this paper we implemented all models using fixed effects for SITE for comparability.

## Results

### Pattern of missingness

No baseline data were missing. 74% of subjects had complete data (costs and EQ-5D) at 1 year, 10% at year 3 and 25% at year 5 (Table 2). This pattern arises from the staggered recruitment and because the final questionnaire was administered at a fixed calendar point irrespective of when the subject was recruited.

**Table 2:** Missing data pattern

Time point	Missing pattern (Costs, EQ-5D)			
	Complete cost and complete EQ5D	Complete cost and missing EQ5D	Missing cost and complete EQ5D	Missing cost and missing EQ5D
At 1-year	74% N = 333	19% N = 85	0.2% N = 1	7% N = 31
At 3-years	9.7% N = 44	74% N = 333	0%	16.3% N = 73
At 5-years	25.3% N = 114	7% N = 31	0%	67.7% N = 305

The logistic model showed the probability that a value is missing in costs and EQ-5D are related to the time in follow-up, age at baseline and site (p < 0.0001), see Additional file 1: Tables S6, S7. As EQ-5D tend to change over time since surgery (see Additional file 1: Table S9), and EQ-5D are more likely to be missing at longer follow-up, this suggests that the probability of an item being missing may be correlated with values of observed outcomes (MAR). However, it cannot be ruled out that data might be MNAR (that is, missingness correlated with unobserved outcomes).

Only subjects with complete aggregate data were used in CCA: year 1, n = 338; year 3, n = 44 and year 5, n = 147. The BPA included all the 450 subjects. The data for RMM and MI included all the longitudinal observations for all follow-ups as an unbalanced panel.

### Cost effectiveness analysis

Table 3 shows a summary of the results of the cost-effectiveness-analysis with the six different approaches at each time point. All methods agreed that there was no statistically significant difference in cost at the 5% level at any time horizon. Early intervention was associated with statistically significantly greater mean QALY among all methods at year 1. BPA showed a statistically significant difference at year 3, while other methods tended towards greater QALY for the intervention, but this did not reach statistical significance.

**Table 3:** Results of the models

	Time point	RMM	RMFE	CCA	MIPMM	MILR	BPA
Differences in mean costs (standard error) (95% confidence interval) (£)	1-year	N = 450 − 70 (482) CI (− 1014 to 874)	N = 450 − 93 (525) CI (− 1123 to 936)	N = 338 − 4 (326) CI (− 644 to 636)	N = 450 50 (295) CI (− 528 to 627)	N = 450 (307) CI (− 534 to 669)	N = 450 137(305) CI (− 340 to 665)
	3-years	N = 450 − 159 (565) CI (− 1265 to 949)	N = 450 − 180 (610) CI (− 1375 to 1015)	N = 44 215 (831) CI (− 1531 to 148)	N = 450 25 (312) CI (− 586 to 637)	N = 450 58 (328) CI (− 583 to 700)	N = 450 − 38 (360) CI (− 637 to 556)
	5-years	N = 450 − 93 (651) CI (− 1369 to 1184)	N = 450 − 111 (697) CI (− 1477 to 1255)	N = 147 464 (751) CI (− 1008 to 1936)	N = 450 8 (333) CI (− 645 to 661)	N = 450 57 (354) CI (− 637 to 751)	N = 450 1200 (807) CI (− 122 to 2536)
Differences mean QALY (standard error) (95% confidence interval)	1-year	N = 450 0.05 (0.02) CI (0.02 to 0.08)	N = 450 0.05 (0.02) CI (0.02 to 0.08)	N = 338 0.04 (0.02) CI (0.01 to 0.07)	N = 450 0.05 (0.02) CI (0.01 to 0.08)	N = 450 0.05 (0.02) CI (0.01 to 0.08)	N = 450 0.05(0.02) CI (0.02 to 0.78)
	3-years	N = 450 0.07 (0.07) CI (− 0.06 to 0.20)	N = 450 0.07 (− 0.07) CI (− 0.06 to 0.20)	N = 44 0.04 (0.13) CI (− 0.21 to 0.29)	N = 450 0.08 (0.05) CI (− 0.04 to 0.20)	N = 450 0.09 (0.08) CI (− 0.07 to 0.25)	N = 450 0.12 (0.13) CI (0.09 to 0.34)
	5-year	N = 450 0.05 (0.11) CI (− 0.16 to 0.26)	N = 450 0.05 (0.11) CI (− 0.16 to 0.26)	N = 147 0.01 (0.12) CI (− 0.24 to 0.25)	N = 450 0.05 (0.08) CI (− 0.10 to 0.20)	N = 450 05 (0.12) CI (− 0.20 to 0.31)	N = 450 0.16 (0.17) CI (− 0.03 to 0.58)
ICER £/QALY	1-year	Dominant	Dominant	Dominant	1082	1430	2728
	3-years	Dominant	Dominant	6075	319	627	Dominant
	5-years	Dominant	Dominant	59,500	159	1010	7394

*RMM* repeated measure mixed model, *RMFE* repeated measure fixed effect, *CCA* complete-case-analysis, *MIPMM* multiple imputation using predictive mean matching, *MILR* multiple imputation using linear regression, *BPA* Bayesian parametric approach, *QALY* quality-adjusted life years, *ICER* incremental cost ratio

**Fig. 2:**
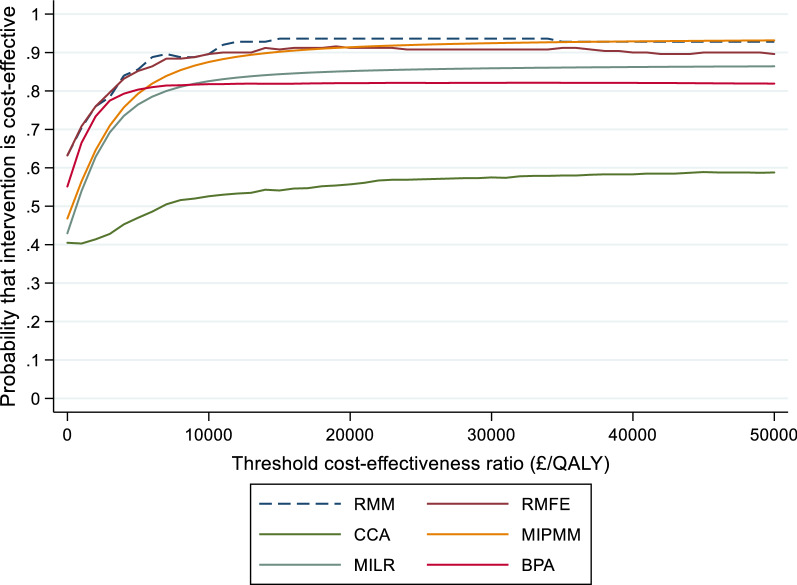
Cost-effectiveness acceptability curves at 3 years. *RMM* Repeated measure mixed model, *RMFE* Repeated measure fixed effect, *MIPMM* multiple imputation using predictive men matching, *MILR* multiple imputation using linear regression, *CCA* complete-case-analysis, *BPA* Bayesian parametric approach

At 3 years early intervention dominated according to RMM, RMFE and BPA methods. The ICER according to CCA was £6075/QALY, £319/QALY using PMM and £627/QALY using MILR. All methods suggested that early intervention is cost-effective at a threshold of £30,000 per QALY at 1-, and 3-year time horizons. At a threshold of £30,000/QALY, the estimated probability that the intervention was cost-effective was 93% using RMM, 91% using RMFE and 58% using CCA, see Fig. 2.

When we compare the two methods for multiple imputation, MIPMM show a loss of efficiency of 0.03% in costs using M = 40 and 0.8% in QALY while MILR shows 0.20% and 1.3% for costs and QALY, respectively. MCE were less than 10% of the standard errors (SE) in both methods, indicating reasonable stability of the models. As would be expected, imputations with MIPMM correspond more closely than MILR to the distribution of observed data (Additional file 1: Fig. S1).

**Fig. 3:**
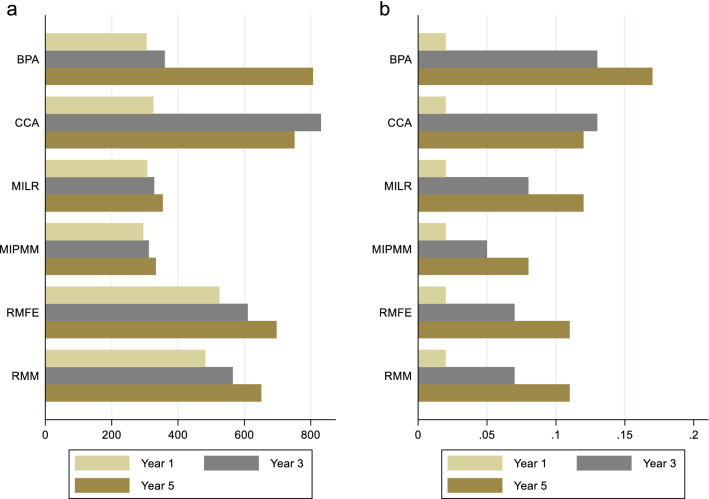
Standard errors of **a** incremental mean costs **b** incremental mean QALY. *RMM* repeated measure mixed model, *RMFE* repeated measure fixed effect, *CCA* complete case-analysis, *MIPMM* multiple imputation using predictive mean matching, *MILR* multiple imputation using linear regression, *BPA* Bayesian parametric approach

RMM and RMFE showed greatest standard errors (SE), 482 and 525, respectively at year 1 for incremental mean costs than other methods (Fig. 3a). CCA showed the greatest SE at year 3 and BPA at year 5, 831 and 807, respectively. MIPMM showed the lowest SE at all time horizons. Regarding QALY at year 1, CCA and BPA showed greater SE than other methods (Fig. 3b). BPA presented the highest SE at year 3 and 5. Other methods showed similar SE for incremental mean QALY at years 1, 3 and 5.

## Discussion

This paper compared six methods for handling missing data empirically, some in common use and others less so, using a real data set with several follow-up points over a long time period. We have attempted to use a similar estimation model in each case, so that differences arise mainly from the number of subjects and observations per subject that comprise the data, and the assumed latent correlation between observed and missing data.

The original cost-effectiveness analysis employed RMM, and reported mean total cost of − £155 (95% CI − £1262 to £953) and mean total QALY of 0.073 (95% CI − 0.06 to 0.20) at 3 years [4]. The very small differences arise in this paper because the original paper coded SITE as a random effect. In this paper, we code SITE as a factor variable (fixed effect). All the approaches coincide in estimating statistically significantly greater QALY at 1 year, but only BPA showed a statistically significant difference in QALY at 3 years. RMM, RMFE, MILR, MIPMM and BPA suggest the mean difference in QALY is positive (in favour of early intervention). However, the mean coefficient for incremental cost is negative in some methods and positive in others, leading to differences in the ICER.

CCA is the simplest method to implement. However, because subjects with any incomplete observations are discarded, it can be considered wasteful of the available data. Hence it is likely that the standard errors are over-estimates, arising from the low number of observations. CCA can also be biased if data are MAR. Hence the ICER for CCA could be inaccurate. Other methods coincide in suggesting that early intervention is cost-effective at a threshold of £30,000 per QALY at 1-, 3- and 5-year time horizons. However, the variation in the ICER across the methods does generate some additional methodological uncertainty, underlining the importance of conducting sensitivity analyses using alternative methods.

BPA offers a principled framework for handling missing data under the assumption of MAR. BPA includes all individuals but uses aggregate data for the dependent variables. This means that if a subject has one missing EQ-5D follow-up, then the QALY for that individual would be recorded as missing, and previous (or future) follow-ups for EQ-5D for that individual would be ignored. This means BPA can also be considered wasteful when (as is the case here) many individuals have some missing EQ-5D, in the sense that some relevant data is ignored. Hence it might be reasonable to conclude that the large standard errors generated by BPA at 3 and 5 years in this example are over-estimates.

MI, RMM and RMFE employ all the available longitudinal period cost and EQ-5D observations in all the subjects. Hence, they can be considered efficient methods in the sense that every item of observed data is used in the analysis model. This is important when there is substantial item missingness, as we have in this dataset. They are straightforward to implement using standard software. RMM and RMFE would not be a suitable option if there were considerable missing baseline covariates that needed to be included in the analysis model (**selection** and CCA share this limitation). There were slight differences between RMM and RMFE. This may be due to the cluster size.

MI has been widely recommended for cost-effectiveness analysis [1, 42–44]. MI can impute both missing outcome data and missing baseline data. Also, simulation studies have found that MIPMM offers a better fit to the data [45]. Some caution is needed when using MIPMM if there are few donors in the vicinity of an incomplete case, leading to a risk of bias [33]. Also, if a donor is selected for many individuals or repeatedly used by the same individual across imputations this will lead to inefficiency, underestimating the between-imputation variance. MI can compute the variance–covariance matrix of total mean cost and total mean QALY using parametric assumptions, while RMM and RMFE estimates costs and EQ-5D separately and uses bootstrap simulations to estimate the correlation between total mean cost and total mean QALY. This makes both RMM and RMFE rather slow to compute, though some analysts may favour semi-parametric methods such as bootstrap when data are not normally distributed.

## Strengths and limitations

This study has compared the missing data approaches reported in Gohel et al. [4] against a wider set of methods for handling missing data. We included approaches that are commonly used, and others less so [1, 9], in a case study with a long follow up and a high proportion of item missingness There are also some limitations that need to be taken into account. First, other missing data approaches are available [46–48]. We only examined MAR mechanisms here. If data are MNAR then this may give rise to bias. The data could have been modelled as a three-level multilevel MI (time, subject and site). When the percentage of missing data is large MI strategies that do not take into account the intra-cluster correlation can underestimate the variance of the treatment effect [7, 49, 50]. Other Bayesian models could also have been tried to model sites as random effects [5, 51]. Also, costs and QALY were assumed normally distributed for the simplicity of modelling [52]. In this case study the standard errors for RM models were generally greater than for MIPMM. However, since we do not know the true values of the missing data, we cannot generalize about which method is “correct”.

## Conclusion

The variation in the results across the methods underline the importance of conducting sensitivity analyses using alternative approaches to missing data. Further work might consider models for handling non-normal distributions and more complex missing data mechanisms.

## Supplementary Information

**Additional file 1. **Further description of methods, data quality and results of regression analyses.

## Data Availability

The datasets used and/or analysed during the current study are available from the corresponding author on reasonable request. Also, codes of STATA and R used in this study are available in Mendeley Data, V1, http://dx.doi.org/10.17632/j8fmdwd4jp.5.
