# Supplementary material for: Comparing methods for handling missing cost and quality of life data in the Early Endovenous Ablation in Venous Ulceration trial
Source: Cost Eff Resour Alloc. 2022 Apr 7;20:18. doi: 10.1186/s12962-022-00351-6 (PMC8991820; doi:10.1186/s12962-022-00351-6)
Supplement: Supplementary file 1 — Additional file 1. Further description of methods, data quality and results of regression analyses. [file 12962_2022_351_MOESM1_ESM.docx]

**Supplementary material**

**Description of the difference between models that use aggregate data and models that use longitudinal data**

We present a hypothetical example to explain the different ways that the models use the available data. Table S1 shows 3 hypothetical stylised subjects, in a dataset with outcome variables for period costs and EQ-5D collected at the end of two time periods (year 1 and year 2). Subject 1 had complete data, subject 2 was missing cost data at follow-up, and subject 3 was missing EQ-5D data at one follow-up. Hence total cost over 2 years cannot be calculated for subject 2 and total QALY cannot be calculated over 2 years for subject 3.

Table S1: Hypothetical dataset with missing data

| Subject ID | Baseline EQ-5D | Costs during year 1 | Costs during year 2 | EQ-5D at the end of year 1 | EQ-5D at the end of year 2 |
| --- | --- | --- | --- | --- | --- |
| 1 | 0.5 | 10 | 15 | 0.5 | 0.5 |
| 2 | 0.6 | missing | 20 | 0.6 | 0.6 |
| 3 | 0.4 | 5 | 10 | missing | 0.4 |

Complete case analysis (CCA) would only include the aggregate observations from subject 1 in the model, and subjects 2 and 3 would be excluded from the analysis (see Table S2).

Table S2: Aggregate data included in CCA

| Subject ID | Total cost over 2 years | Total QALY over 2 years |
| --- | --- | --- |
| 1 | 25 | 1 |
| 2 | Subjects 2 and 3 are excluded from the CCA analysis | |
| 3 |  |  |

Bayesian Parametric Analysis (BPA) would include aggregate data for all 3 subjects as shown in Table S3. The missing data the total cost for subject 2 and the total QALY for subject 3 would be estimated as parameters by the software.

Table S3: Aggregate data included in BPA

| Subject ID | Total cost up to the end of year 2 | Total QALY up to the end of year 2 |
| --- | --- | --- |
| 1 | 25 | 1 |
| 2 | missing | 1.2 |
| 3 | 15 | missing |

Multiple Imputation (MI) using chained equations would include all the longitudinal data from Table S1. MI imputes the missing data at each interim period and generates M complete data sets. Total cost and total QALY are then calculated passively for each imputed dataset for all 3 subjects. Both methods (MILR and MIPMM) described in the main paper allow the imputation. Total mean cost and total mean QALY are then estimated jointly for each imputed dataset using bivariate normal regression, and estimates are combined across the imputed datasets using Rubin’s rules. The cost-effectiveness acceptability curve can be estimated parametrically from the variance-covariance matrix of the regression.

Table S4: Disaggregate data included in MI

| Subject ID | Total cost up to the end of year 2 | Total QALY up to the end of year 2 |
| --- | --- | --- |
| 1 | 25 | 1 |
| 2 | missing | 1.2 |
| 3 | 15 | missing |

Choosing an imputation model

MILR: $Y_{i} \sim N(\beta_{0}+\beta_{1}X_{,}, \sigma^{2}$)

1. Estimate $\beta=(\beta_{0,} \beta_{1}, \sigma)$ from complete cases, giving estimates $\hat{\beta_{0,}} \hat{\beta_{1}},\hat{\sigma}$
2. Draw $\beta$ from its posterior

- For linear regression this done exactly
- Giving perturbed parameters ${\beta^{*}}_{0}+ {\beta^{*}}_{1}$and $\sigma^{*}$

1. Impute missing values for subject 2 and 3. Here involves ${Y^{*}}_{i}\sim N({\beta^{*}}_{0}+ {\beta^{*}}_{1} X_{i},{\sigma^{*}}^{2})$

b) and c) are repeated m times to create m datasets

MIPMM:

1. Regress $Y$ on $X$ using cases with $Y$ observed, giving $\hat{\beta}$
2. Draw $\beta^{*}$
3. Impute all the missing values of $Y$:

- Predict all observed $Y$ values using $\hat{\beta}$
- Predict all missing $Y$ values using $\beta^{*}$
- For each missing $Y$value:

1. Find the $K$ individuals with observed $Y$ whose $\hat{\beta}X$ are nearest to $\beta^{*}X$ for the missing value
2. Select one of these $K$ individual at random
3. Impute using the chosen individual’s value of $Y$

The repeated measures mixed model (RMM) and fixed effect (RMFE) also includes all the longitudinal data from Table S1. RMM and RMFE estimate separate models for the period costs and EQ-5D. All the available data (the period costs at each time point, and the EQ-5D at each time point) are used in the analysis model, and then the total mean costs and total mean QALY are predicted from the estimated coefficients. Bootstrap can be used to estimate the correlation between total mean cost and total mean QALY.

**Data quality**

Table S5. Data quality

| Did the trial involve a quality manager? | Trial manager |
| --- | --- |
| Measures taken to anticipate the extent, pattern, and causes of missing data at the study design stage | A follow-up on any missing data from the outset to keep to a minimum |
| Design of cost components of the questionnaires | The resource diary was designed in collaboration with the Health Economist and trial project team, based on previous experience |
| How were questionnaires piloted? | Patient resource use diaries were used in other vascular studies by this team with minor adaptions for this context |
| Use of measures for improving probabilities that respondents would answer the phone | Cost data was collected at the same time of clinical data so repeated calls were made if the phone was not answered. Interviewers would note convenient times to call individual participants |
| Did any data collection coordination exist between phone calls and photos? | Yes, the interviewers would be aware when the ulcer was healed or not and therefore could prompt the participants re: expected resource use |
| How were interviewers selected and trained? | The interviewers were Vascular nurses, trained by the Trial Manager at the outset and on an ongoing basis when required |
| Did data collectors use paper forms or enter data directly into an electronic device? | Paper forms were used which were transcribed into the electronic database. A subset of forms at each recruiting site were checked annually for data entry errors |
| Were random checks performed on completed forms and data completeness, accuracy, and consistency? | Reports were run on a bimonthly basis by the Trial Manager to review data completeness, accuracy, and consistency. |
| Did interviewers meet regularly with supervisors for feedback, discussion of quality control procedures, and observation checks? | The trial manager regularly fed back any observations from the data reviews, or monitoring visits to ensure consistency of the data collection between the research sites |
| Were proportions of missing data across interviewers compared? | No, but each site received feedback to ensure consistency between sites |
| Measures in response to detected errors | Data queries were raised in the database and followed up until completion or exhaustion of efforts. |

Supplementary figure 1. Distribution of imputations at 3-year for MILR and MIPMM

MILR: Multiple imputation using linear regression MIPMM multiple imputation using predictive mean matching

Table S6: Missingness mechanism in costs

Table S7: Missingness mechanism in EQ5D

Table S8. Treatment costs over 1 to five years using repeated measures fixed effect.

Table S9. EQ-5D over 1 to 5 years using repeated measures fixed effect.
